# AI’s pivotal impact on redefining stakeholder roles and their interactions in medical education and health care

**DOI:** 10.3389/fdgth.2024.1458811

**Published:** 2024-11-05

**Authors:** Jayne S. Reuben, Hila Meiri, Hadar Arien-Zakay

**Affiliations:** ^1^ Texas A&M School of Dentistry, Dallas, TX, United States; ^2^ Department of Surgery, Cedars-Sinai Medical Center, Los Angeles, CA, United States; ^3^ Department of Surgery, Sheba Tel-Hashomer Medical Center, Associated with Tel-Aviv University, Tel-Aviv, Israel; ^4^ The Faculty of Medicine, School of Pharmacy, Institute for Drug Research, The Hebrew University of Jerusalem, Jerusalem, Israel

**Keywords:** medical education, Artificial Intelligence (AI), machine learning, healthcare, strategies and guidelines, clinical decision support systems, ethics

## Abstract

Artificial Intelligence (AI) has the potential to revolutionize medical training, diagnostics, treatment planning, and healthcare delivery while also bringing challenges such as data privacy, the risk of technological overreliance, and the preservation of critical thinking. This manuscript explores the impact of AI and Machine Learning (ML) on healthcare interactions, focusing on faculty, students, clinicians, and patients. AI and ML's early inclusion in the medical curriculum will support student-centered learning; however, all stakeholders will require specialized training to bridge the gap between medical practice and technological innovation. This underscores the importance of education in the ethical and responsible use of AI and emphasizing collaboration to maximize its benefits. This manuscript calls for a re-evaluation of interpersonal relationships within healthcare to improve the overall quality of care and safeguard the welfare of all stakeholders by leveraging AI's strengths and managing its risks.

## 1 Introduction

Artificial Intelligence (AI) offers numerous opportunities to revolutionize medical training, diagnostics, treatment planning, and healthcare delivery through the development of computer systems that can perform tasks traditionally requiring human intelligence, such as decision-making, language understanding, and pattern recognition (1). For example, AI has been utilized in diagnostic imaging to assist radiologists in identifying patterns in medical images (2). Within the realm of AI, Machine Learning (ML) is a critical subset in which algorithms learn from data, improving their accuracy and effectiveness over time. This self-improving capability is particularly well-suited for applications in medicine, such as predicting patient outcomes based on electronic health record data, where models can become more accurate as they process more information over time (3).

Large Language Models (LLMs), for instance, have transformed natural language processing (NLP), leading to advanced AI chatbots that are capable of offering precise, context-aware responses. LLMs represent a significant leap in AI's ability to understand and generate human language, enabling more natural and effective interactions between machines and users. These systems may utilize a public or private knowledge base to provide accurate answers to user inquiries on medical issues (4, 5). For example, with an estimated 100 million weekly users, ChatGPT can potentially enhance human diagnostic performance through collective intelligence. Collective intelligence refers to AI's ability to aggregate and analyze vast amounts of data from diverse sources, including clinical studies, case reports, and real-time user interactions. This aggregation allows for a more comprehensive understanding of complex medical conditions, potentially leading to more accurate and timely diagnosis (2, 6–8). Another example is the profound potential of AI to enhance medical training by simulating patient scenarios, such as virtual patients with varying symptoms, allowing medical trainees to practice and refine their diagnostic and treatment skills in a risk-free, feedback-driven environment (9). In healthcare delivery, AI and ML streamline operations, improve efficiency, and reduce costs. For instance, AI algorithms can optimize appointment scheduling by predicting no-show rates, integrating them into electronic health records to facilitate better data management, and analyzing patient data to identify those at risk of developing certain conditions (10–12).

However, the use of AI-based technologies in medical settings also raises significant concerns regarding ethics and drawbacks that should be carefully addressed (10). These concerns include data privacy, validation, bias, and fairness, in which flawed algorithms could worsen disparities in treatment across demographic groups. For instance, if AI systems are trained on biased data, they might inadvertently recommend less effective treatments for minority populations, thereby exacerbating the existing health disparities. To address this, several frameworks have been proposed to mitigate bias and ensure equitable benefits for all patient populations (11, 12). Lack of transparency in AI decisions can undermine trust, making it difficult for clinicians to validate AI-generated recommendations and resulting in hesitation regarding their use. Just as important, the vast amount of data utilized by AI raises significant privacy and security concerns, with risks of data breaches or misuse, which could lead to unauthorized access to sensitive patient information (13, 14). Accountability and liability are also challenging as it remains unclear who should be held responsible when AI errors occur. Finally, the ability of AI to generate hallucinations, seemingly credible but incorrect or fabricated information, can lead to clinical inaccuracies if not properly monitored (13). This risk is particularly pronounced when non-clinicians use clinical decision support systems (CDSS) (15) without adequate expertise. Although AI integration into simulations and virtual patients can be valuable for skill development, it may lack real-world variability and fail to develop vital communication skills, requiring extensive faculty and clinician's oversight. Ethical concerns, such as emotional intelligence deficits, educational inequities, and the potential for plagiarism, also arise with AI's integration into medical education, requiring careful consideration and policy development at the institutional level.

Therefore, medical education faces a pivotal choice on how to actively integrate AI into clinical training for its safe use or to allow external influences to determine its role (16). A timely integration of AI is essential to ensure that medical education equips future clinicians with the necessary tools for success. To adjust to the fast-growing user base, evaluating AI's impact on healthcare education requires thorough and iterative examinations of its influence on the communication patterns of four key populations-faculty, students, clinicians and patients. This study explores the influence of AI on each of these stakeholders and the changing communication patterns it introduces, and proposes strategies and guidelines for effective AI pedagogical integration in healthcare education.

## 2 AI's multifaced impact on medical education, practice, and care

### 2.1 AI's influence on faculty

The integration of AI in healthcare education is poised to transform teaching, assessment, and content creation for faculty (17, 18). AI-driven virtual assistants can offer personalized guidance and automate assessments, whereas content-generation AI in healthcare education tools streamlines the creation of engaging materials (19). While AI can improve creativity and productivity, faculty will need to adjust the quality and complexity of assessments as AI can easily be used to answer questions at lower Bloom's levels (remember, understand, apply) and even higher (analyze, evaluate, create) levels (20). Bloom's Taxonomy is a hierarchical model that categorizes educational objectives, with lower levels involving basic recall of facts (remember), understanding concepts, and applying knowledge to straightforward scenarios (21). AI excels at these lower levels because it can rapidly retrieve, process, and apply information using advanced algorithms and data analyses. This capability necessitates a shift in strategies to include assessments that critically evaluate AI-generated outputs as well. Several software products for learning and competency assessments are already available and are used in a wide range of fields in the corporate world for applicant hiring and employee evaluations (22, 23). Similar platforms are already being used in medical education for student and faculty evaluations to assess writing and detect AI-generated content, indicating that the integration of these technologies into educational evaluations is underway (24). The expectation is that this trend will continue to grow, leading to the adoption of even more sophisticated AI-driven assessment tools in medical education to enhance the evaluation process for both faculty and students.

These tools may also result in a broader evaluation portfolio, in which all three learning domains-cognitive, affective, and psychomotor-can be more readily assessed (24–26), including not only knowledge and comprehension of learned materials but also student engagement, analysis of complex situations, critical thinking, creativity, teamwork, and reflection. Furthermore, evaluation of programmatic elements may include student survey data and satisfaction, quality of learning materials, course structure, curriculum, innovative learning methods, and department involvement. In terms of faculty benefits, AI can rapidly reduce the time required to complete several tasks. For example, it can significantly streamline the evaluation process, allowing more efficient grading and feedback. AI tools can automatically grade assessments, analyze student engagement through learning analytics, and provide insight into how students interact with course materials. This allows faculty to focus more on mentoring and supporting students rather than administrative tasks. AI can also assist faculty in identifying students who may be at risk of falling behind by analyzing patterns in attendance, participation, and assignment completion, thus enabling timely interventions. Moreover, faculty benefit from AI's ability to evaluate the effectiveness of programmatic elements, such as course structure and curriculum design, by analyzing student survey data, satisfaction scores, and overall engagement, which can inform continuous improvement efforts.

Faculty development in medical education can be supported by AI-based digital solutions, offering clinical decision support systems (CDSS) to educators, thus enhancing their ability to rapidly create clinical scenarios for learning (15). These AI-driven tools provide educators with up-to-date medical knowledge, patient data analysis, and evidence-based guidelines, enabling them to create more dynamic and clinically relevant learning experiences. For example, using CDSS, faculty can simulate complex patient cases and use diagnostic recommendations, thus demonstrating the application of theory to practice. CDSS can offer real-time feedback on diagnosis or treatment choices, allowing faculty to identify gaps in understanding and adjust their teaching strategies effectively. However, one should keep in mind that this brings new challenges, including hallucinations, information bias, and a lack of transparency (12, 13). AI hallucinations occur when LLMs generate seemingly credible but incorrect or fabricated information, often linked to information bias and exacerbated by a lack of transparency in how AI processes and presents clinical data. This mandates that users rely on peer-reviewed, evidence-based CDSS platforms (27) to avoid low-quality outputs, which may be generated when using widely utilized AI platforms such as LLMs. However, even with peer-reviewed and evidence-based systems, the risk of hallucinations can be decreased but is still present. In addition, the use of evidence-based CDSS by non-clinicians may result in clinical inaccuracy owing to a lack of expertise in prompt engineering and an inability to interpret the AI output to identify these hallucinations and challenge any information bias that may be present.

Another example of AI integration is the use of simulations and virtual (computer-generated) patients, which are valuable for skill development in medical education (9). These tools allow learners to interact with simulated clinical scenarios, practicing decision-making and treatment planning in a risk-free environment while replicating various medical conditions. However, virtual patients may lack real-world variability, which can limit the scope of learning, and often focus on technical skills, neglecting vital communication abilities such as empathy and patient interaction. Additionally, these systems may be resource-intensive to maintain (28).

Therefore, faculty expertise coupled with extensive, iterative development remains essential for facilitating in-depth discussions and deep learning. In this approach, AI can be used as a complementary partner rather than a replacement for faculty expertise. A faculty that can effectively harness AI's capabilities will enhance the learning environment by integrating AI into areas such as real-time feedback and personalized learning, while still leading the critical aspects of clinical reasoning and communication. Coordinated collaboration between educators and clinicians, providing both realistic scenarios in preclinical medical education and creating a common language among stakeholders, is therefore highly recommended. This integration ensures that AI supports and amplifies faculty contributions, rather than substituting them.

Additional ethical concerns and data privacy issues should be addressed. Emotional intelligence deficits, educational inequities, academic integrity, and copyright ownership are some of issues that may arise with AI integration (13). University leadership as well as academic policy makers are now challenged with laying out the foundations for AI in education. Committees evaluating appropriate integration and usage of AI should be established at the institutional level to define specific guidelines and recommendations but may need refinement according to individual departments and courses. To this end, numerous universities have already implemented guidelines and policies for the use of AI. This topic is further explored in the manuscript under the section “Strategies and Guidelines for AI Pedagogical Integration in Healthcare Education”.

### 2.2 Empowering students through AI

Rote memorization and lecture-based learning have lost their roles as the main components of medical education (21, 29–32). In contrast, AI offers students personalized enhanced learning experiences through intelligent tutoring systems (33), reflective self-assessments (34), virtual laboratories and immersive simulations (35). Strategies for efficient and responsible AI use and interpretation, as well as the identification of pitfalls, bias, and errors, should be integrated into modern curricula. This will provide students with the necessary skill sets for current and future AI platforms. Examples include incorporating case studies where students must analyze AI-generated diagnoses for bias, using AI in simulations for decision-making practice, providing exercises that highlight common AI errors and limitations, teaching students to apply AI tools critically in clinical settings. We also recommend educating students on the capabilities and limitations of CDSS, limiting their use where foundational skills need to be developed and encouraging their use for knowledge integration in clinical scenarios. AI integration also provides rapid access to data and knowledge, leading to more equitable global opportunities (36). Peer-to-peer learning may also be facilitated by AI, which can foster collaboration and community among students to match participants, identify resources, and enable worldwide knowledge exchange. However, AI may have the opposite effect and potentially minimize team engagement if learners opt to forgo working with others. Additional concerns with AI include the collection and utilization of student data, raising valid privacy and accuracy concerns, student overreliance on AI technology, ease of plagiarism, and potential lack of critical thinking development. Therefore, attention to these potential drawbacks should be addressed in curricular discussions.

### 2.3 AI in clinical practice for enhanced patient care

AI has the potential to revolutionize healthcare by improving the quality, safety, and efficiency of medical treatment. Based on patient medical records, history, genetics, risk factors, medications, and preferences, more rapidly obtained algorithms may also offer prediction of conditions (37–40) as well as precision treatment plans (6, 7, 41). Furthermore, novel treatments can be suggested for patients fitting the inclusion criteria for clinical trials by using AI to analyze medical records, genetic data, and trial databases, matching patients with trials based on their conditions and biomarkers. Medical databases and peer-reviewed frontier information will be more concise and accessible, allowing clinicians to stay current in their field. In addition, AI's role as a gatekeeper in patient safety is becoming more eminent. For example, alerts for potential complications during invasive procedures, life-threatening situations, and drug-related issues may provide better medical quality and protection against human errors, delayed diagnoses, or misdiagnoses (42–45). Moreover, AI's influence extends beyond clinical care and advances the optimization of the healthcare workflow. By streamlining administrative tasks, such as appointment scheduling, billing, and record-keeping, AI can allow healthcare providers to allocate more time to patient care (46–49).

Yet with these improvements, important limitations of AI must be noted. These include data bias at multiple levels, lack of consideration for socioeconomic determinants of health, patient preferences, religious or cultural beliefs, and direct clinical observations (50). The inability to incorporate or even recognize the influence of these variables could exacerbate health disparities. Also, given the sensitive nature of patient data, privacy issues and informed consent must also be considered, as well as the cost of constantly evolving technology in healthcare systems with potential technical issues that disrupt workflow.

### 2.4 Improved patient advocacy and health literacy through AI

The use of AI can transform patient ownership by providing platforms that help individuals better understand and manage their health. These platforms can continuously track patients’ biometric data, sending personalized notifications that prompt timely actions and ensure adherence to treatment plans. For example, some platforms aim to better control chronic diseases, such as glycemic control, through ongoing monitoring and AI-based alerts (51), patient adherence with automatic scheduling (48), and drug management reminders (52). NLP and chatbots provide patients with unprecedented access and interpretation to understand complex medical information (52, 53). AI-facilitated telemedicine may offer immediate healthcare guidance and assistance to patients to prevent disease or manage progression (54). AI-powered health apps and platforms offer comprehensive medical information that promotes health literacy, whereas real-time monitoring and virtual assistants may ensure continuous feedback and treatment adherence. Yet, there should be considerable apprehension about patients’ capacity to discern between trustworthy and misleading online health data and the potential for misinterpretation (52). In our view, these future medical AI-platforms for patients should be based on peer-reviewed databases, include scaffoldings and structured templates to guide the patients in their use. These platforms will provide access to information, references, and specific explanations relevant to the patient's specific conditions, and should be integrated with real-time expert validation to minimize the risk of misinformation. To avoid inadequate self-treatment, these platforms will also direct the patient to the relevant clinician and suggest further activities that needs to be done. This is an extremely important feature as patients may over-rely on AI for medical guidance, potentially leading to a lack of a needed interaction with expert healthcare professionals. Moreover, patient security, privacy, and confidentiality concerns emerge as personal health data is shared with AI systems. These concerns include the risk of unauthorized access, data breaches, and the misuse of sensitive health information. AI systems must follow strict data protection regulations, implement strong encryption, ensure transparency in data usage, and give patients control over their information to address privacy concerns (14).

## 3 AI in healthcare: redefining relationships, roles, and education

**Figure 1:**
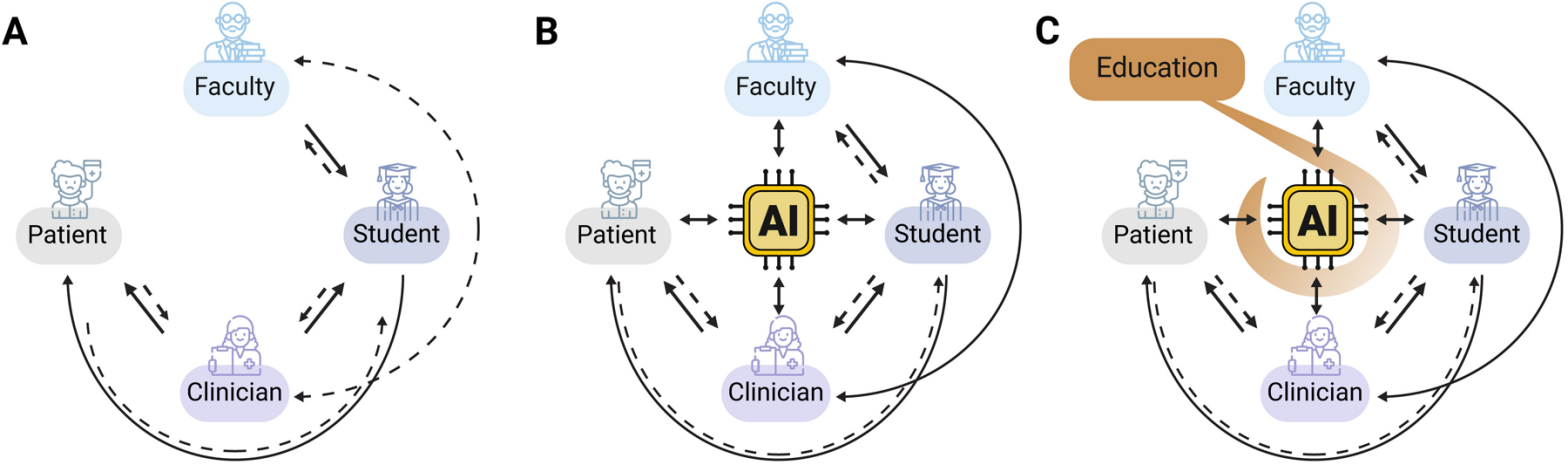
Changes in key health system players’ interrelationships in the era of AI. The arrows symbolize the dynamic interactions between the four stakeholders, with the AI positioned at the center as a new participant. Solid lines represent a higher level of information access and input; dashed lines represent a relatively lower level of access and input. **(A)** Before the availability of AI technologies, communication primarily followed a bidirectional pattern with patients being relatively passive recipients of information and instructions. Students constructed knowledge and gained competencies under the guidance of faculty members and clinicians, progressively developing their expertise in problem-solving and patient treatment. The health system's responsibility was primarily centered on clinicians and patients, with faculty involvement in preclinical education. Faculty and clinician professionals collaborated to reduce the gap between education and clinical practice. **(B)** With the availability of AI technologies to patients, students, and experts, medical knowledge is now more accessible to all stakeholders. This shifts communication interrelationships towards a more multi-directional approach, breaking down language and competency barriers and making AI a collaborative partner in decision-making. **(C)** Incorporation of AI education and training plays a crucial role in guiding all players for the effective and responsible use of AI, maintaining the desired level of expertise to minimize misinterpretations and medical errors.

The assimilation of AI into healthcare enterprises dramatically empowers each of the four populations, thus changing the nature of their involvement in medical processes and consequently shifting the pivot point in their relationships (Figure 1). For many years, health professionals, who served as educators and practitioners, were at the core of healthcare, imparting knowledge and skills through a patriarchal, primarily linear pattern. Students, the future healthcare workforce, developed under the guidance of educators and clinicians in this model. Patients were mostly on the receiving end and afforded minimal input for their own health care. This predominantly unidirectional model has already significantly changed to a bidirectional model (Figure 1A). In an AI-facilitated reality, an additional transformative shift occurs in these interactions, moving to a multi-directional network. As a partner for learning, data collection, and problem solving, AI can effectively narrow the language and knowledge gaps between professional and non-expert stakeholders by translating technical jargon into lay language at the specific educational level of the patient or student (Figure 1B). Therefore, educating all four populations on the responsible use of AI in medicine regarding its opportunities and potential dangers is imperative (Figure 1C). Effective communication requires faculty members and clinicians to be adept at working with the learning tools and knowledge platforms used by the new generation of students and be able to adapt them for improved teaching and learning. Clinicians and residents should receive training in communicating with patients who are turning to AI for answers, thus balancing the risks associated with self-treatment due to AI while continuing to encourage patients to be more involved in their healthcare decisions (Figure 1C).

## 4 Stakeholder interactions in AI-facilitated health

AI can enhance patient-provider communication by providing personalized health information, reminders, and follow-up recommendations. For instance, chatbots and asynchronous online information about the exact procedures and processes involved can answer common patient queries reducing the burden on providers. Thus, these AI platforms could be accessed by patients at their convenience. AI-driven appointment scheduling can screen and clear patients for appointments, optimize patient wait times, improve access, and reduce administrative overheads. Predictive AI analytics can help allocate resources efficiently. However, a decrease in the humanistic element and potential misuse of patient data by AI could potentially affect empathy and patient trust. Therefore, balancing AI use with human communication is crucial to meet the unique needs of each patient.

AI can assist medical students in learning by providing real-time information during patient encounters. Enhanced virtual simulations can reinforce clinical skills training. However, the sole use of AI could result in less exposure to diverse patient cases, limit hands-on experiences, and spread misinformation. To mitigate this potential adverse effect, AI platforms should be trained on reliable validated data from diverse patient populations.

For faculty and clinicians, AI can support the creation of personalized learning paths for students, adapting to their needs and progress. These paths coupled with expert mentorship will prevent students from missing out on nuanced clinical insights that may not be captured by these platforms. In addition, AI can help facilitate collaboration amongst providers and streamline referrals to reduce the patient time spent waiting to see a specialist. While AI can rapidly analyze medical literature and data to assist in evidence-based decisions, overreliance could limit creativity and ingenuity. For example, in a power outage or cyber-attack, the health care provider must still be able to function and respond appropriately with limited equipment or resources.

## 5 Strategies and guidelines for AI pedagogical integration in healthcare education

To ensure a thorough and practical understanding of AI and ML in healthcare, early and continued incorporation into medical training is crucial. Therefore, we advocate for making AI and ML fundamental to the medical curriculum through specialized courses during preclinical (55, 56) and clinical (57) years, as well as integration into postgraduate and continuing medical education. For instance, cardiovascular disease modules could include discussions on current and emerging AI tools for imaging and patient monitoring (58). For this to occur, AI will also need to be included in accreditation standards and board exams. Recognizing that early-stage medical students might initially struggle to see the relevance of a technologically driven subject, leveraging student-centered methods, such as problem-based learning (PBL) (59) and AI-incorporated case studies, will enhance motivation and mastery of the AI language (19). Hands-on AI projects and simulations can provide practical experiences, whereas discussions and reflections on the ethical use of AI in medicine can broaden students’ understanding of its wider implications. The challenge will be to create authentic simulations that mimic actual practice.

Courses and workshops should aim to equip medical practitioners with the skills to navigate both medical and technological dialogues effectively, understand AI's potential as well as discern inappropriate technologies for their needs, and evaluate the accuracy and limitations of AI analyses (60). This requires specialized training for the faculty and clinicians, which extends beyond traditional medical education. Workshops, seminars, conferences, and continuing education (CME) modules for AI and ML applications in education and medicine will need to contain the technical aspects, ethical considerations, data privacy, and guidance on the interpretation of AI-generated data (60). For example, a workshop series could cover topics ranging from basic AI concepts and tools to advanced subjects, such as interpreting AI images and research findings.

To this end, many universities have already established guidelines and policies for AI use. For example, the Cornell University task force created reports for using generative AI in teaching, research, and administration (61). Stanford University has created several modules that faculty can access for help with integrating AI into their courses (62). The University of Helsinki (63), and the University of Waterloo (64) guidelines allow course coordinators/directors to decide the extent in which AI can be used. Duke University has also launched a website solely dedicated to AI use in healthcare in addition to its general university guidelines (65–67). The University of Oxford has created guidelines for their students (68), as well as an institute for the ethical use of AI (69).

For clinicians, CME credits in AI applications and hands-on training with AI-powered tools, such as Google's DeepMind AI or RETFound (70) for eye disease detection, may emphasize the practical integration and interpretation of AI recommendations. In addition, clinicians should be educated on communicating insights to patients while also focusing on the aforementioned limitations and risks of AI, including potential biases, as well as the importance of maintaining a critical perspective on AI-generated data. Collaboration among educators, medical practitioners, biomedical researchers, and computer scientists through a shared language can also inspire the development of innovations that meet the evolving needs of the healthcare sector. The World Health Organization (WHO) has released two documents that summarize the risks for LMM use in healthcare, as well as regulatory and ethical considerations (71, 72) and could be used as the basis for these discussions. In addition, the American Medical Association (AMA) has published several articles for AI in medicine (73). For referencing the European Union's stance on AI in healthcare, the 2022 report by the European Parliamentary Research Service is a valuable source. It discusses the role of AI in enhancing clinical practice while focusing on compliance with regulations like General Data Protection Regulation (GDPR) (74), alongside addressing concerns about data privacy, security, and ethical use (14). Table 1 provides a summary of recommendations for clinicians from these and other references listed in this paper.

**Table 1:** Recommendations for clinicians Use of AI.

For All Clinicians	
Stay informed: standards and guidelines	Familiarize yourself with key regulatory frameworks and guidelines such as the EU AI Act (75, 76), the Blueprint for an AI Bill of Rights (USA) (77), the WHO (71, 72), the AMA (73), and other relevant organizations in their own country or countries of practice.
	Regularly review updates on AI regulations and guidelines to stay current with best practices and compliance requirements, such as those provided by the FDA (78), including the Proposed Regulatory Framework for Modifications to AI/ML-Based Software as a Medical Device (SaMD) (79) and Artificial Intelligence and Medical Products: How CBER, CDER, CDRH, and OCP are Working Together (80).
Stay informed: updated tools	Define clinical aspects where AI tools can be incorporated in your practice (e.g., drug recommendations and interactions, follow-up pending results, radiological diagnostics, etc.)
	Decide which AI platforms are most appropriate for your needs by considering those vetted by organizations such as ORCHA, Happtique, and the UK NHS App Certification Programme (81)
	Stay updated with upcoming platforms and clinical trials and ensure the AI tools have undergone rigorous validation and testing to minimize biases and errors, regulatory approval status, transparency in data usage, and documented performance metrics.
Data privacy and security	Implement robust data protection measures to safeguard patient information, in compliance with legal and ethical standards.
	Ensure AI tools maintain the confidentiality of patient data by adhering to privacy regulations, including the EU's GDPR (74) and HIPAA in the USA (82).
Broad AI applications	Define areas where AI tools can be incorporated in your practice to improve patient outcomes and streamline practice management (e.g., administrative work, scheduling, patient compliance, patient satisfaction, telemedicine platforms).
	Gradually incorporate AI tools in the various areas while evaluating their effectiveness and upgrade as novel tools developed.
Patient education	Educate patients about the benefits and limitations of AI tools in their care.
	Ensure informed consent is obtained when using AI tools in patient care.
Professional development	Enroll in continuous education, online courses and certifications on AI in healthcare offered by reputable institutions (e.g., CME credits).
	Join professional organizations and stay updated on specialty-specific AI advancements through journals, conferences, and professional networks.
Consult with experts	Seek advice from AI experts and consultants to evaluate and implement AI tools effectively.
	Collaborate with other healthcare providers and systems to share insights and experiences with AI applications.
For Clinicians Associated with a University	
Collaborate with research departments	Leverage academic resources and connect with institutional IT support to stay updated on AI advancements, ensuring seamless access to tools, training, and technical assistance for enhanced healthcare applications.
	Engage with university research departments to access cutting-edge AI tools and participate in clinical trials.
	Encourage collaboration with university's computer science experts to develop novel AI platforms for specific needs arising from the medical field.
Educational initiatives	Contact institutional centers for teaching and learning to access workshops on AI use.
	Consult with faculty development leaders to provide educational programs and workshops on AI in healthcare.
	Encourage interdisciplinary collaboration to integrate AI knowledge across different medical specialties.
For Hospital settings	
Specialized AI tools	Identify AI tools tailored to your specialty, such as radiology, surgery, oncology, or cardiology, and evaluate their effectiveness and regulatory compliance, according to standards like the FDA's Proposed Regulatory Framework for AI/ML-Based SaMD (79) and the European Parliamentary Research Service report on AI in healthcare (14).
	Participate in specialty-specific AI research and development projects to contribute to the advancement of AI in your field.
Multidisciplinary team work	Utilize AI platforms that support an environment of collaborative communication among all providers in the care team.
	Incorporate AI platforms that improves care providers administrative management and reduces unnecessary paper work.

AMA, American Medical Association; CBER, Center for Biologics Evaluation and Research; CDER, Center for Drug Evaluation and Research; CDHR, Center for Devices and Radiological Health; CME, Continuing Medical Education; EU, European Union; FDA, United States Food and Drug Administration; GDPR, General Data Protection Regulation; HIPAA, Health Insurance Portability and Accountability Act; IT, Information Technology; NHS, National Health Service; OCP, Office of Combination Products; ORCHA, Organization for the Review of Care and Health; WHO, World Health Organization.

Professional guidance of patients by medical staff is crucial to effectively communicate the benefits and risks of AI and ML in healthcare (53). Patients could benefit from educational campaigns aimed at improving AI health literacy and explaining how AI tools enhance their care. Transparent communication should also include the limitations of these technologies to help build trust and empower patients to make informed decisions regarding their health. One recommendation is to create a smart-phone medical AI application with embedded prompt engineering scaffolds for patients (as well as providers and students) that only utilizes peer-reviewed, validated evidence and data. The application output could be reviewed together with the provider. An additional innovation would be the creation of programs that train medical personnel with expertise in patient care and AI. Examples of these unique health care providers exist at the Sheba Medical Center in Tel Aviv, Israel. There, AI usage in the emergency medical department provides easy, fast and reliable medical care for patients with limited face-to-face interaction while still leveraging the expertise of trained AI medical professionals (unpublished).

## 6 Conclusion

AI will continue to redefine professional health education and patient care. This transformation in healthcare necessitates a re-evaluation of interpersonal connections and relationships making it imperative to educate all involved groups on AI's responsible use in health care. The integration of AI can support faculty in providing personalized education, equipping students with advanced learning tools, enhancing clinician opportunities for better, more rapid data-driven decisions, and empowering patients to advocate for their health. In this manner, AI users will be more aware of the limitations of the systems that they access, so that AI's strengths can be leveraged to enhance the quality of care, while also safeguarding those involved from its potential risks.

## Data Availability

The original contributions presented in the study are included in the article/Supplementary Material, further inquiries can be directed to the corresponding author.

## References

[1] Alowais SA Alghamdi SS Alsuhebany N Alqahtani T Alshaya AI Almohareb SN Revolutionizing healthcare: the role of artificial intelligence in clinical practice. BMC Med Educ. (2023) 23:689. 10.1186/s12909-023-04698-z 37740191 PMC10517477

[2] Wu S-H Tong W-J Li M-D Hu H-T Lu X-Z Huang Z-R Collaborative enhancement of consistency and accuracy in US diagnosis of thyroid nodules using large language models. Radiology. (2024) 310:e232255. 10.1148/radiol.232255 38470237

[3] Esteva A Chou K Yeung S Naik N Madani A Mottaghi A Deep learning-enabled medical computer vision. NPJ Digit Med. (2021) 4:5. 10.1038/s41746-020-00376-2 33420381 PMC7794558

[4] Thirunavukarasu AJ Ting DSJ Elangovan K Gutierrez L Tan TF Ting DSW. Large language models in medicine. Nat Med. (2023) 29:1930–40. 10.1038/s41591-023-02448-8 37460753

[5] Tayebi Arasteh S Han T Lotfinia M Kuhl C Kather JN Truhn D Large language models streamline automated machine learning for clinical studies. Nat Commun. (2024) 15:1603. 10.1038/s41467-024-45879-8 38383555 PMC10881983

[6] Rodman A Buckley TA Manrai AK Morgan DJ. Artificial intelligence vs clinician performance in estimating probabilities of diagnoses before and after testing. JAMA Netw Open. (2023) 6:e2347075. 10.1001/jamanetworkopen.2023.47075 38079174 PMC10714249

[7] Fonseca Â Ferreira A Ribeiro L Moreira S Duque C. Embracing the future—is artificial intelligence already better? A comparative study of artificial intelligence performance in diagnostic accuracy and decision-making. Eur J Neurol. (2024) 31:e16195. 10.1111/ene.16195 38235841 PMC11235701

[8] Cil G Dogan K. The efficacy of artificial intelligence in urology: a detailed analysis of kidney stone-related queries. World J Urol. (2024) 42:158. 10.1007/s00345-024-04847-z 38483582 PMC10940482

[9] Hamilton A. Artificial intelligence and healthcare simulation: the shifting landscape of medical education. Cureus. (2024). 16:e59747. 10.7759/cureus.59747 38840993 PMC11152357

[10] Lee P Bubeck S Petro J. Benefits, limits, and risks of GPT-4 as an AI chatbot for medicine. N Engl J Med. (2023) 388:1233–9. 10.1056/NEJMsr2214184 36988602

[11] Abràmoff MD Tarver ME Loyo-Berrios N Trujillo S Char D Obermeyer Z Considerations for addressing bias in artificial intelligence for health equity. NPJ Digit Med. (2023) 6:170. 10.1038/s41746-023-00913-9 37700029 PMC10497548

[12] Nazer LH Zatarah R Waldrip S Ke JXC Moukheiber M Khanna AK Bias in artificial intelligence algorithms and recommendations for mitigation. PLOS Digit Health. (2023) 2:e0000278. 10.1371/journal.pdig.0000278 37347721 PMC10287014

[13] Zhui L Fenghe L Xuehu W Qining F Wei R. Ethical considerations and fundamental principles of large language models in medical education: viewpoint. J Med Internet Res. (2024) 26:e60083. 10.2196/60083 38971715 PMC11327620

[14] European Parliamentary Research Service. Artificial intelligence in healthcare. (2022). chrome-extension://efaidnbmnnnibpcajpcglclefindmkaj/ Available online at: https://www.europarl.europa.eu/RegData/etudes/STUD/2022/729512/EPRS_STU(2022)729512_EN.pdf (accessed October 18, 2024).

[15] Sutton RT Pincock D Baumgart DC Sadowski DC Fedorak RN Kroeker KI. An overview of clinical decision support systems: benefits, risks, and strategies for success. NPJ Digit Med. (2020) 3:17. 10.1038/s41746-020-0221-y 32047862 PMC7005290

[16] Cooper A Rodman A. AI and medical education — a 21st-century pandora’s box. N Engl J Med. (2023) 389:385–7. 10.1056/NEJMp2304993 37522417

[17] Zuckerman M Flood R Tan RJB Kelp N Ecker DJ Menke J ChatGPT for assessment writing. Med Teach. (2023) 45:1224–7. 10.1080/0142159X.2023.2249239 37789636

[18] Laupichler MC Rother JF Grunwald Kadow IC Ahmadi S Raupach T. Large language models in medical education: comparing ChatGPT- to human-generated exam questions. Acad Med. (2023) 99:508–12. 10.1097/ACM.0000000000005626 38166323

[19] Divito CB Katchikian BM Gruenwald JE Burgoon JM. The tools of the future are the challenges of today: the use of ChatGPT in problem-based learning medical education. Med Teach. (2024) 46:320–2. 10.1080/0142159X.2023.2290997 38149617

[20] Giffin SD. A taxonomy of internet applications for project management communication. Project Manag J. (2002) 33:39–47. 10.1177/875697280203300405

[21] Arien-Zakay H. Blended learning in nursing pharmacology: elevating cognitive skills, engagement and academic outcomes. Front Pharmacol. (2024) 15:1361415. 10.3389/fphar.2024.1361415 38455960 PMC10917888

[22] Madanchian M Taherdoost H Mohamed N. AI-based human resource management tools and techniques; A systematic literature review. Procedia Comput Sci. (2023) 229:367–77. 10.1016/j.procs.2023.12.039

[23] Kelly J. How Companies Are Hiring And Reportedly Firing With AI. Available online at: https://www.forbes.com/sites/jackkelly/2023/11/04/how-companies-are-hiring-and-firing-with-ai/ (accessed October 18, 2024).

[24] Bloom BS Krathwohl DR. Taxonomy of Educational Objectives; the Classification of Educational Goals by a Committee of College and University Examiners. Handbook I: Cognitive Domain. New York, NY: Longmans, Green (1956).

[25] Krathwohl DR Bloom BS Masia BB. Taxonomy of Educational Objectives: The Classification of Educational Goals, Hand Book II: Affective Domain. New York: David Mckay Company In corporated (1964).

[26] Harrow AJ. A Taxonomy of the Psychomotor Domain: A Guide for Developing Behavioral Objectives. New York: David McKay (1972).

[27] Ng K Kartoun U Stavropoulos H Zambrano JA Tang PC. Personalized treatment options for chronic diseases using precision cohort analytics. Sci Rep. (2021) 11:1139. 10.1038/s41598-021-80967-5 33441956 PMC7806725

[28] Kononowicz AA Woodham LA Edelbring S Stathakarou N Davies D Saxena N Virtual patient simulations in health professions education: systematic review and meta-analysis by the digital health education collaboration. J Med Internet Res. (2019) 21:e14676. 10.2196/14676 31267981 PMC6632099

[29] Li Z Li F Fu Q Wang X Liu H Zhao Y Large language models and medical education: a paradigm shift in educator roles. Smart Learn Environ. (2024) 11:26. 10.1186/s40561-024-00313-w

[30] Mbakwe AB Lourentzou I Celi LA Mechanic OJ Dagan A. ChatGPT passing USMLE shines a spotlight on the flaws of medical education. PLOS Digit Health. (2023) 2:e0000205. 10.1371/journal.pdig.0000205 36812618 PMC9931307

[31] Araújo B Gomes SF Ribeiro L. Critical thinking pedagogical practices in medical education: a systematic review. Front Med (Lausanne). (2024) 11:1358444. 10.3389/fmed.2024.1358444 38947238 PMC11211358

[32] Lewis KO Popov V Fatima SS. From static web to metaverse: reinventing medical education in the post-pandemic era. Ann Med. (2024) 56:2305694. 10.1080/07853890.2024.2305694 38261592 PMC10810636

[33] Turner L Hashimoto DA Vasisht S Schaye V. Demystifying AI: current state and future role in medical education assessment. Acad Med. (2023) 99:S42–7. 10.1097/ACM.0000000000005598 38166201

[34] Turner L Weber DE Santen SA Olex AL Baker P Overla S Making use of natural language processing to better understand medical students’ self-assessment of clinical skills. Acad Med. (2024) 99:285–9. 10.1097/ACM.0000000000005527 37976396 PMC10922291

[35] Reed JM Dodson TM. Generative AI backstories for simulation preparation. Nurse Educ. (2023) 49: 184–8. 10.1097/NNE.0000000000001590 38151702

[36] Liu X Wu J Shao A Shen W Ye P Wang Y Uncovering language disparity of ChatGPT on retinal vascular disease classification: cross-sectional study. J Med Internet Res. (2024) 26:e51926. 10.2196/51926 38252483 PMC10845019

[37] Wei Q Xiao Y Yang T Chen J Chen L Wang K Predicting autism spectrum disorder using maternal risk factors: a multi-center machine learning study. Psychiatry Res. (2024) 334:115789. 10.1016/j.psychres.2024.115789 38452495

[38] Yang C Huebner ES Tian L. Prediction of suicidal ideation among preadolescent children with machine learning models: a longitudinal study. J Affect Disord. (2024) 352:403–9. 10.1016/j.jad.2024.02.070 38387673

[39] Mehrabi Nasab E Sadeghian S Vasheghani Farahani A Yamini Sharif A Masoud Kabir F Bavanpour Karvane H Determining the recurrence rate of premature ventricular complexes and idiopathic ventricular tachycardia after radiofrequency catheter ablation with the help of designing a machine-learning model. Regen Ther. (2024) 27:32–8. 10.1016/j.reth.2024.03.001 38496010 PMC10940794

[40] Lin S Lu W Wang T Wang Y Leng X Chi L Predictive model of acute kidney injury in critically ill patients with acute pancreatitis: a machine learning approach using the MIMIC-IV database. Ren Fail. (2024) 46:2303395. 10.1080/0886022X.2024.2303395 38264967 PMC10810629

[41] Moor M Banerjee O Abad ZSH Krumholz HM Leskovec J Topol EJ Foundation models for generalist medical artificial intelligence. Nature. (2023) 616:259–65. 10.1038/s41586-023-05881-4 37045921

[42] Carroll J Colley E Cartmill M Thomas SD. Robotic tomographic ultrasound and artificial intelligence for management of haemodialysis arteriovenous fistulae. J Vasc Access. (2023):11297298231210019. 10.1177/11297298231210019 37997016 PMC11849249

[43] Wang B Yu J Lin S Li Y Huang W Yan S Intraoperative AI-assisted early prediction of parathyroid and ischemia alert in endoscopic thyroid surgery. Head Neck. (2024) 46:1975–87. 10.1002/hed.27629 38348564

[44] Chen C Chen Y-L Scholl J Yang H-C Li Y-CJ. Ability of machine-learning based clinical decision support system to reduce alert fatigue, wrong-drug errors, and alert users about look alike, sound alike medication. Comput Methods Programs Biomed. (2024) 243:107869. 10.1016/j.cmpb.2023.107869 37924770

[45] Gallo RJ Shieh L Smith M Marafino BJ Geldsetzer P Asch SM Effectiveness of an artificial intelligence–enabled intervention for detecting clinical deterioration. JAMA Intern Med. (2024) 184:557–62. 10.1001/jamainternmed.2024.0084 38526472 PMC10964159

[46] Young RA Martin CM Sturmberg JP Hall S Bazemore A Kakadiaris IA What complexity science predicts about the potential of artificial intelligence/machine learning to improve primary care. J Am Board Family Med. (2024) 37:332–45. 10.3122/jabfm.2023.230219R1 38740483

[47] Mohanasundari SK Kalpana M Madhusudhan U Vasanthkumar K BR Singh R Vashishtha N Can artificial intelligence replace the unique nursing role? Cureus. (2023) 15:e51150. 10.7759/cureus.51150 38283483 PMC10811613

[48] Knight DRT Aakre CA Anstine C V Munipalli B Biazar P Mitri G Artificial intelligence for patient scheduling in the real-world health care setting: a metanarrative review. Health Policy Technol. (2023) 12:100824. 10.1016/j.hlpt.2023.100824

[49] Blezek DJ Olson-Williams L Missert A Korfiatis P. AI Integration in the clinical workflow. J Digit Imaging. (2021) 34:1435–46. 10.1007/s10278-021-00525-3 34686923 PMC8669074

[50] Suresh H Guttag J. A Framework for Understanding Sources of Harm Throughout the Machine Learning Life Cycle. Equity and Access in Algorithms, Mechanisms, and Optimization. New York, NY, USA: ACM (2021). p. 1–9. 10.1145/3465416.3483305

[51] Guan Z Li H Liu R Cai C Liu Y Li J Artificial intelligence in diabetes management: advancements, opportunities, and challenges. Cell Rep Med. (2023) 4:101213. 10.1016/j.xcrm.2023.101213 37788667 PMC10591058

[52] Gordon EB Towbin AJ Wingrove P Shafique U Haas B Kitts AB Enhancing patient communication with chat-GPT in radiology: evaluating the efficacy and readability of answers to common imaging-related questions. J Am Coll Radiol. (2024) 21:353–9. 10.1016/j.jacr.2023.09.011 37863153

[53] Shahsavar Y Choudhury A. User intentions to use ChatGPT for self-diagnosis and health-related purposes: cross-sectional survey study. JMIR Hum Factors. (2023) 10:e47564. 10.2196/47564 37195756 PMC10233444

[54] Sharma S Rawal R Shah D. Addressing the challenges of AI-based telemedicine: best practices and lessons learned. J Educ Health Promot. (2023) 12:338. 10.4103/jehp.jehp_402_23 38023098 PMC10671014

[55] Park SH Pinto-Powell R Thesen T Lindqwister A Levy J Chacko R Preparing healthcare leaders of the digital age with an integrative artificial intelligence curriculum: a pilot study. Med Educ Online. (2024) 29:2315684. 10.1080/10872981.2024.2315684 38351737 PMC10868429

[56] Zhao C Xu T Yao Y Song Q Xu B. Comparison of case-based learning using Watson for oncology and traditional method in teaching undergraduate medical students. Int J Med Inform. (2023) 177:105117. 10.1016/j.ijmedinf.2023.105117 37301132

[57] Abid A Murugan A Banerjee I Purkayastha S Trivedi H Gichoya J. AI education for fourth-year medical students: two-year experience of a web-based, self-guided curriculum and mixed methods study. JMIR Med Educ. (2024) 10:e46500. 10.2196/46500 38376896 PMC10915728

[58] Alnasser TN Abdulaal L Maiter A Sharkey M Dwivedi K Salehi M Advancements in cardiac structures segmentation: a comprehensive systematic review of deep learning in CT imaging. Front Cardiovasc Med. (2024) 11:1323461. 10.3389/fcvm.2024.1323461 38317865 PMC10839106

[59] Trullàs JC Blay C Sarri E Pujol R. Effectiveness of problem-based learning methodology in undergraduate medical education: a scoping review. BMC Med Educ. (2022) 22:104. 10.1186/s12909-022-03154-8 35177063 PMC8851721

[60] Doherty G McLaughlin L Hughes C McConnell J Bond R McFadden S. A scoping review of educational programmes on artificial intelligence (AI) available to medical imaging staff. Radiography. (2024) 30:474–82. 10.1016/j.radi.2023.12.019 38217933

[61] IT@Cornell. Cornell’s AI strategy. Available online at: https://it.cornell.edu/ai (accessed October 18, 2024).

[62] Stanford University. Artificial Intelligence Teaching Guide. Available online at: https://teachingcommons.stanford.edu/teaching-guides/artificial-intelligence-teaching-guide (accessed October 18, 2024).

[63] University of Helsinki. Artificial Intelligence in Teaching. https://teaching.helsinki.fi/instructions/article/artificial-intelligence-teaching (accessed October 18, 2024).

[64] University of Waterloo. UW Course Outline Suggestions for Generative Artificial Intelligence. https://uwaterloo.ca/associate-vice-president-academic/uw-course-outline-suggestions-generative-artificial (accessed October 18, 2024).

[65] Duke University. AI Health. https://aihealth.duke.edu/ (accessed October 18, 2024).

[66] Duke University. Learning Innovation and Lifetime Education; Artificial Intelligence Policies: Guidelines and Considerations. https://lile.duke.edu/ai-and-teaching-at-duke-2/artificial-intelligence-policies-in-syllabi-guidelines-and-considerations/ (accessed October 18, 2024).

[67] Duke University. Learning Innovation and Lifetime Education; Generative AI and Teaching. https://lile.duke.edu/ai-and-teaching-at-duke-2/ (accessed October 18, 2024).

[68] University of Oxford. Use of generative AI tools to support learning. https://www.ox.ac.uk/students/academic/guidance/skills/ai-study (accessed October 18, 2024).

[69] Oxford University. Institute for Ethics in AI. https://www.oxford-aiethics.ox.ac.uk/ (accessed October 18, 2024).

[70] Zhou Y Chia MA Wagner SK Ayhan MS Williamson DJ Struyven RR A foundation model for generalizable disease detection from retinal images. Nature. (2023) 622:156–63. 10.1038/s41586-023-06555-x 37704728 PMC10550819

[71] World Health Organization. Regulatory considerations on artificial intelligence for health. License: CC BY-NC-SA 3.0 IGO. (2023) Available online at: https://www.who.int/publications/i/item/9789240078871 (accessed October 18, 2024).

[72] World Health Organization. Ethics and governance of artificial intelligence for health: guidance on large multi-modal models. License: CC BY-NC-SA 3.0 IGO. (2024) Available online at: https://www.who.int/publications/i/item/9789240084759 (accessed October 18, 2024).

[73] American Medical Association. Augmented intelligence in medicine. (2024) https://www.ama-assn.org/practice-management/digital/augmented-intelligence-medicine (accessed October 18, 2024).

[74] European Union. General Data Protection Regulation (GDPR) (EU) 2016/679. (2016) Available online at: https://eur-lex.europa.eu/legal-content/EN/TXT/?uri=CELEX%3A32016R0679 (accessed October 18, 2024).

[75] Busch F Kather JN Johner C Moser M Truhn D Adams LC Navigating the European union artificial intelligence act for healthcare. NPJ Digit Med. (2024) 7:210. 10.1038/s41746-024-01213-6 39134637 PMC11319791

[76] European Commission. Proposal for a Regulation Laying Down Harmonized Rules on Artificial Intelligence (Artificial Intelligence Act) and Amending Certain Union Legislative Acts. (2021) Available online at: https://eur-lex.europa.eu/legal-content/EN/TXT/?uri=CELEX%3A52021PC0206 (accessed October 18, 2024).

[77] The White House Office of Science and Technology Policy. Blueprint for an AI Bill of Rights: Making Automated Systems Work for the American People. (2022) Available online at: https://www.whitehouse.gov/ostp/ai-bill-of-rights/ (accessed October 18, 2024).

[78] U.S. Food and Drug Administration. Artificial Intelligence and Machine Learning in Software as a Medical Device. https://www.fda.gov/medical-devices/software-medical-device-samd/artificial-intelligence-and-machine-learning-software-medical-device (accessed October 18, 2024).

[79] U.S. Food and Drug Administration. Proposed Regulatory Framework for Modifications to Artificial Intelligence/Machine Learning (AI/ML)-Based Software as a Medical Device (SaMD). chrome-extension://efaidnbmnnnibpcajpcglclefindmkaj/ (2019) Available online at: https://www.fda.gov/media/122535/download (accessed October 18, 2024).

[80] U.S. Food and Drug Administration. Artificial Intelligence & Medical Products: How CBER, CDER, CDRH, and OCP are Working Together. chrome-extension://efaidnbmnnnibpcajpcglclefindmkaj/ (2024) Available online at: https://www.fda.gov/media/177030/download?attachment (accessed October 18, 2024).

[81] Fitzpatrick PJ. Improving health literacy using the power of digital communications to achieve better health outcomes for patients and practitioners. Front Digit Health. (2023) 5:1264780. 10.3389/fdgth.2023.1264780 38046643 PMC10693297

[82] U.S. Department of Health and Human Services. Health Insurance Portability and Accountability Act (HIPAA). (1996). Available online at: https://www.hhs.gov/hipaa/for-professionals/index.html (accessed October 18, 2024).

